# Short-term effects of single-dose chloral hydrate on neonatal auditory perception: An auditory event-related potential study

**DOI:** 10.1371/journal.pone.0212195

**Published:** 2019-02-08

**Authors:** Qinfen Zhang, Hongxin Li, Xuan Dong, Wenjuan Tu

**Affiliations:** Changzhou Children's Hospital, Changzhou, Jiangsu, PR China; Kochi University of Technology, JAPAN

## Abstract

**Objective:**

To study the short-term effects of a single-dose chloral hydrate on neonatal auditory perception by measuring auditory event-related potentials (aERPs).

**Methods:**

Thirty-nine full-term neonates, aged 2–28 days and weighing 2980–4350 g, were divided into two groups including a chloral hydrate group (CH group, n = 17) and a non-chloral hydrate control group (non-CH group, n = 22). The CH group was given single-dose chloral hydrate (30 mg/kg) orally before aERPs measurement. An auditory oddball paradigm was used to elicit aERPs. P2 and N2 components of the ERP were recorded from electrodes at the Fz and Cz locations, and the areas under their curves (P2 and N2 areas) were calculated for the comparison between two groups.

**Results:**

Significant differences was found in the P2 area between the two groups at Fz and Cz (Fz: F (1,37) = 487.75, *P* < 0.05; Cz: F (1,37) = 1465.94, *P* < 0.05). Similarly, significant difference was also in the N2 area between the two groups at both locations (Fz: F(1,37) = 153.38, *P* < 0.05; Cz: F(1,37) = 798.42, *P* < 0.05).

**Conclusion:**

A single-dose of chloral hydrate impacts neonatal auditory perception in the short-term. Long-term effects will also be studied in future.

## 1. Introduction

Chloral hydrate (CH) is a hypnotic sedative drug widely used in recent years to sedate children[1]. Although its exact mechanism of action remains uncertain, CH is metabolized into trichloroethanol, which is highly lipid soluble and can enter the central nervous system to enable rapid induction of sleep[2]. The half-life of trichloroethanol is 8–12 h in preschoolers, but can be up to four times longer in newborns and preterm infants[3,4].

The widespread use of CH as a sedative can be attributed to its historical safety record. Although CH has been deemed safe within certain parameters including dosage and setting[5], concerns about its safety remain, particularly with regard to potential neurotoxic side effects[4,6,7]. CH has been shown to induce neuroapoptosis in neonatal rats[8]. Several studies have reported the potential of sedation in neonatal and pediatric intensive care patients to be neurotoxic[9,10]. However, prolonged sedation was not shown to be associated with poor neurological outcomes in premature infants when sedation was achieved via mechanical ventilation[11]. A similar report examining the effects of perioperative sedatives on pediatric cardiac surgery did not find an association between the dose and duration of sedatives, and adverse neurodevelopmental outcomes at 18–24 months[12]. CH is widely used in clinic, so it is necessary to further determine its safety. Thus, it is urgent to investigate the neurocognitive effects of CH, especially in newborn patients.

Here, we applied behavioral or quantitative methods to assess the neurocognitive effects of CH. Event-related potentials (ERP) is a well-known cognitive technology. Cognition in neonatal period can induce auditory event-related potentials because of hearing's early development. Auditory event-related potentials (aERPs) reflect neuronal processing and resolution of sound stimuli. This measure provides objective and quantitative data that can be obtained noninvasively. aERPs can thus be used for testing cognitive function early in life, even in newborns whose auditory perception develops by the last 3 months of gestation[13,14]. In this study, aERPs were analyzed to determine if auditory perception in neonates is altered by CH.

## 2. Participants and methods

### 2.1. Participants

CH has been used widely in our department before August 2015. Its use has declined since then following increased awareness of its potential adverse effects, and by January 2017, CH was no longer used in our department. Participants were divided into two groups: CH and non-CH. aERPs were recorded from all participants. The CH group (n = 17) comprised all full-term neonates undergoing CH sedation at the Department of Newborns, Changzhou Children’s Hospital (affiliated with Nantong University, China) between December 2013 and August 2015. The non-CH group (n = 22) comprised full-term neonates who did not receive CH. The aERPs data and medical records of both groups were reviewed and compared. aERPs were recorded when infants were between 2 and 28 days old. CH neonates were given a single-dose oral CH (30 mg/kg) before aERPs measurement. CH was administered by a nurse who had a background in post anesthesia recovery or experience with oral sedation. Non-CH neonates were only provided with baby formula and soothed.

As a retrospective study, all neonates were rigorously screened for normal neonatal cognition. Inclusion criteria included the absence of brain injury during the perinatal period, a neonatal behavioral and neurological assessment (NBNA) score >37 points, good hearing in both ears, stable vital signs, and no obvious organic diseases. Exclusion criteria included neonatal encephalopathy, intracranial hemorrhage, severe hyperbilirubinemia, craniofacial malformation, congenital brain abnormalities, or genetic or metabolic diseases. Neonates were excluded if they had any of the above diseases or symptoms.

The aERPs recordings and procedures were approved by the ethics committee of Changzhou Children’s Hospital. Written informed consent for the aERPs testing was obtained from the guardians of each neonate before the experiment.

### 2.2. ERP recording

ERP recordings were made using a digital 32-channel electroencephalogram (EEG) recording apparatus (Stellate Systems Inc., Quebec, Canada). Recording electrodes were located according to the International 10–20 system, with reference electrodes placed at the ear lobes. ERP data were continuously acquired with a 0.53–35 Hz bandpass and sampled at 500 Hz.

### 2.3. Experimental paradigm

An auditory oddball paradigm was used to generate aERPs. Target stimuli were 2000-Hz tone bursts and non-target stimuli 1000-Hz tone bursts, each lasting for 100 ms with a 1500-ms inter-stimulus interval. Target or non-target stimuli appeared randomly, with target frequency being 10% and non-target frequency being 90%. All stimuli were presented at a sound pressure level of 50 dB. Stimulus presentation was controlled using E-Prime 2.0 software (Psychology Software Tools, Pittsburgh, PA, USA).

### 2.4. Paradigm conditions

The laboratory was sound insulated, with the temperature set at 24–26°C. Neonates were positioned on a comfortable bed with a speaker placed 15 cm away from each ear. After feeding, electrodes (Fz, Cz, F3, F4, C3, and C4) were positioned without using tranquillizers on the heads of the neonates and sound stimuli were administered.

### 2.5. Data collection and analysis

BESA software (MEGIS Software Co., Munich, Germany) was used to calculate the waveform areas for the P2 and N2 components of the aERPs responses to target stimuli at Cz and Fz. The area of waveform refers to the area between the waveform and baseline. If a waveform drifted or was difficult to determine, the time of the component on the average aERPs waveform was used as the time window for measurement. As aERPs waveforms for neonates are irregular, peaks cannot be accurately identified, making it difficult to measure the time between stimulus onset and the onset of the wave ridge. Latency was therefore not assessed in this study.

### 2.6. Statistical analysis

Analyses were performed using SPSS for windows, version 19.0. Statistical significance was set at *P* < 0.05. A one-way ANOVA was conducted to compare P2/N2 areas between the CH and non-CH groups.

## 3. Results

### 3.1. Baseline characteristics of the study population

No patients quit or were excluded from the study, and complete data were obtained from 39 full-term neonates. All these neonates had a gestational age between 39 and 40 weeks and weighed 2980–4350 g. The two groups did not significantly differ in gestational age, age after birth, weight, sex, head circumference or NBNA score (see Table 1).

**Table 1:** Baseline characteristics of the study population.

Newborn Characteristic	CH group	Non-CH group	P-value
Sex-male n(%)	8(47.06)	12(45.45)	0.65
Birth gestation age-weeks(SD)	39.40(0.70)	39.60(0.35)	0.57
Birth weight-kg(SD)	3.32(0.23)	3.41(0.35)	0.37
Head circumference-cm(SD)	34.6(1.05)	34.4(1.20)	0.53
NBNA score(SD)	38.75(0.50)	38.10(0.19)	0.95
Age at visit-days(SD)	16.35(7.08)	15.41(6.72)	0.67
Weight at visit-kg(SD)	3.68(2.05)	3.65(1.87)	0.67

### 3.2. P2 areas differed between CH and non-CH neonates

**Fig 1:**
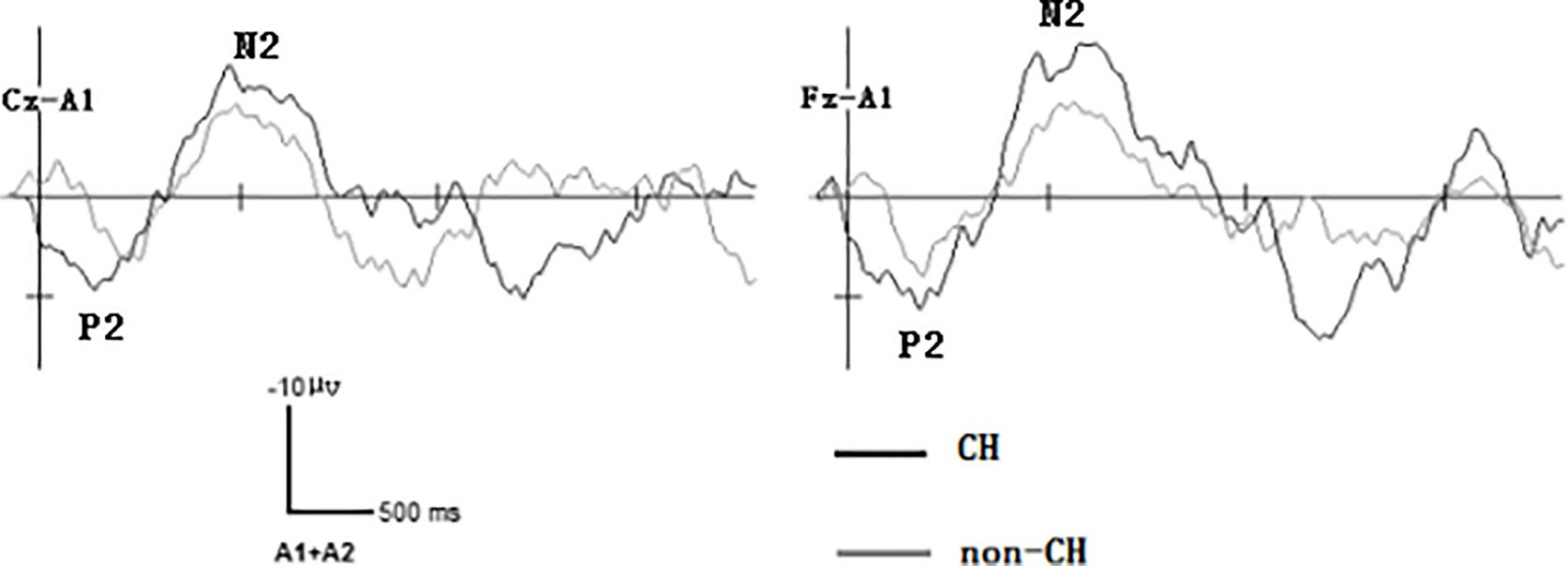
Neonatal grand averages of event-related potentials at Cz and Fz leads.

**Fig 2:**
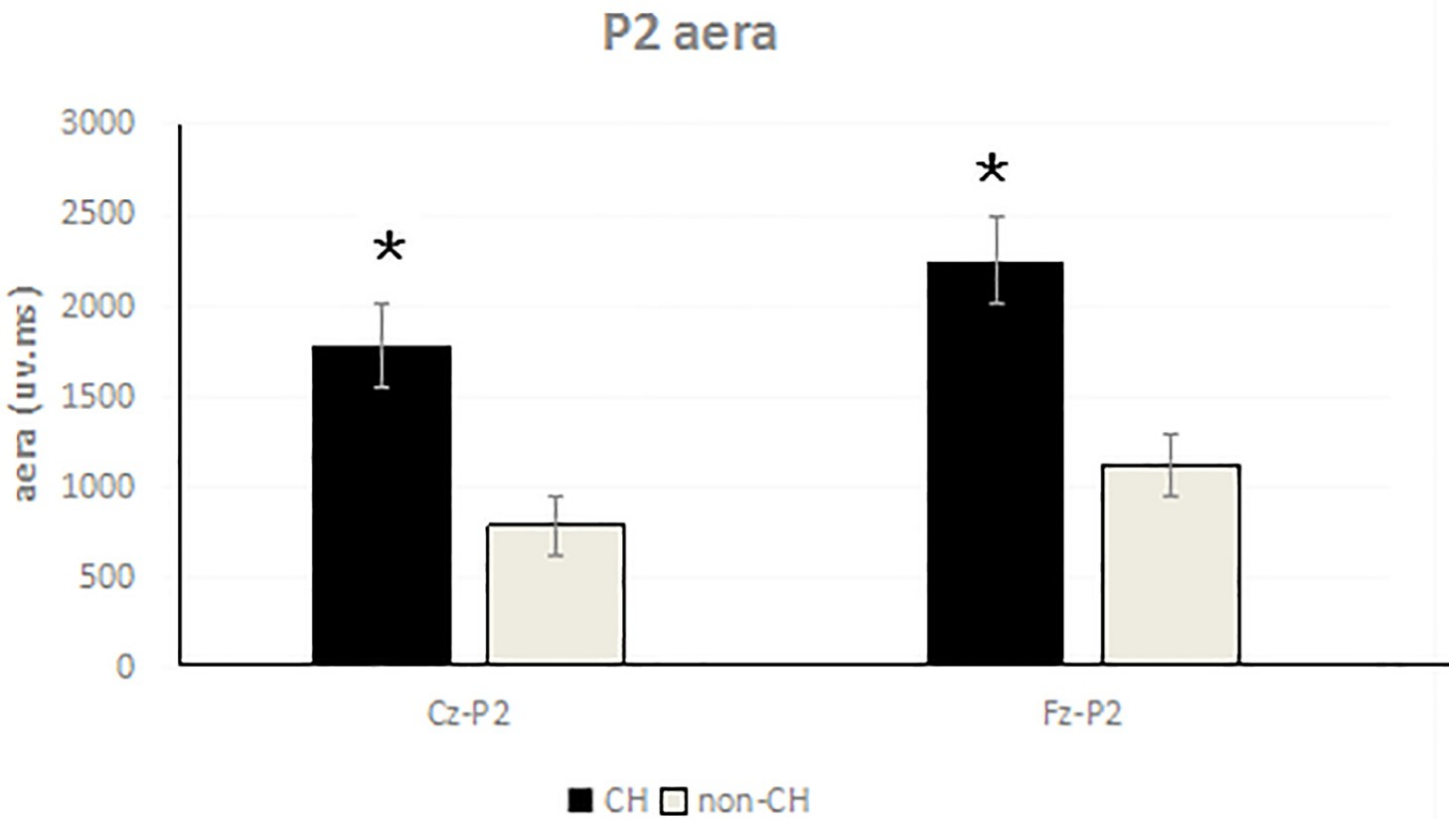
Comparison of P2 areas of CH and non-CH neonatal brain at Cz, Fz leads (P < 0.05).

Mean P2 areas in response to target stimuli were 2249.77 ± 214.35 ms•μv (Fz) and 1782.04 ± 88.70 ms•μv (Cz) for CH neonates and 1121.81 ± 95.23 ms•μv (Fz) and 781.07 ± 74.52 ms•μv (Cz) for non-CH neonates. Analysis revealed that these waveform areas significantly differed between the two groups at both locations. (Fz: F(1,37) = 487.75, *P* < 0.05; Cz: F(1,37) = 1465.94, *P* < 0.05; Figs 1 and 2).

### 3.3. N2 areas differed between CH and non-CH neonates

**Fig 3:**
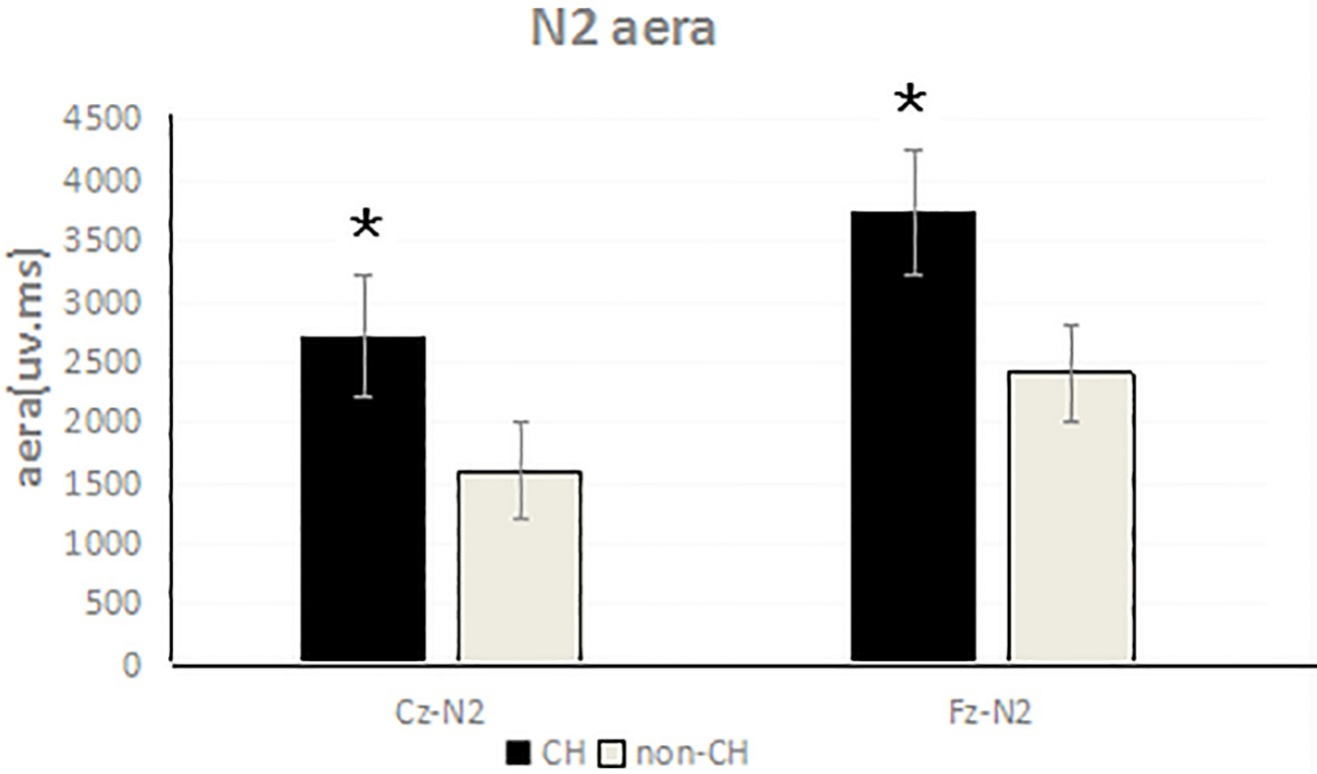
Comparison of N2 areas of CH and non-CH neonatal brain at Cz, Fz leads (P < 0.05).

Mean N2 areas in response to target stimuli were 3747.25 ± 420.12 ms•μv (Fz) and 2718.14 ± 172.20 ms•μv (Cz) for CH neonates and 2412.86 ± 248.35 ms•μv (Fz) and 1607.86 ± 76.07 ms•μv (Cz) for non-CH neonates. Analysis revealed that these waveform areas significantly differed between the two groups at both locations (Fz: F(1,37) = 153.38, *P* < 0.05; Cz: F (1,37) = 798.42, *P* < 0.05; Figs 1 and 3).

## 4. Discussion

Event-related potentials (ERPs) reflect neuronal activity in the brain which is related to cognitive processes. Because of its high temporal resolution, this approach is a powerful tool to study the timing of cognitive processes non-invasively and passively. Thus, it helps to describe the neurobiological basis of cognitive processing in children[15]. The P2 and N2 components of the aERPs are thought to represent the activation of auditory cortical neurons primarily concerned with the perception of sound[16].

The P2 wave is an exogenous ERP component that is related to early information processing and the suppression of irrelevant information[17], as well as attention that is paid to perceptual processing. The N2 wave primarily reflects the psychological process of identifying a target stimulus[18], and is related to the advanced functions such as alertness, making judgments, behavior and cognitive control[19]. Therefore, exploring the P2 and N2 components in response to sound is important in neonates who have experienced chloral hydrate.

The P2 and N2 components represent the activation of auditory cortical neurons after sensory stimulation with sound[16], and the areas under their curves indicate the sum of the electrical potentials for the neurons used in processing the information. We found that both neonatal P2 and N2 areas increased significantly after use of chloral hydrate. So when dealing with the same auditory information, newborns in CH group showed more neurons mobilization, more energy consumption and less efficiency.

As P2 has been suggested to measure sound discrimination[20,21], our results indicated that newborns sedated with chloral hydrate showed a weaker ability to identify the target sound. P2 has also been suggested to measure attention-modulated processes[21,22]. Therefore, our findings might also indicate that these infants likely use more attentional resources in processing target sounds during the early phase of cognitive processing. Thus, the efficiency of early auditory perceptual processing in newborns is reduced after with sedation with a single-dose of chloral hydrate.

As N2 primarily reflects psychological processes related to identifying target stimuli, and because they are closely related to cognitive processes[23,18], this result indicates that the process of identifying auditory information was abnormal, mobilization of neurological resources increased, and information-processing capacity decreased significantly. Thus, a single dose of chloral hydrate can inhibit neonatal brain function by affecting the perception of auditory information and its processing. Stefan et al.[24] reported that the MMN was markedly reduced during deep propofol sedation and indicated that the auditory sensory memory was impaired. These results indicated the effects of sedative drugs on auditory cognition.

The neonatal period is a critical period for cognitive development. Exposure to varying drugs and stressors (painful stimuli, maternal deprivation, hypoglycemia, hypoxia, or ischemia) during this critical period may induce neurodegeneration. It appears that newly born neurons are most vulnerable to the neuroapoptotic (cell death) effects of some sedative drugs[25,26]. Drugs that are routinely utilized to sedate pediatric patients have neurotoxic properties. In laboratory rodent and monkey models, perinatal exposure to sedative drugs leads to neuroapoptosis and subsequent neurocognitive deficits[27,28]. It should be noted that plasticity in the developing neonatal brain is very strong and might drive the recovery from sedation-induced damage.

This study suggests that even if given only a single dose of chloral hydrate, newborns will manifest delayed auditory perception during this vulnerable developmental period.

## 5. Limitation

Our study has some limitations. First, this is a single center retrospective non-randomized study. Second the effect of chloral hydrate is short-term, and hence the long-term effects in later aERPs studies is one of the research contents in future.

## Supporting information

S1 Data P2/N2 areas at Cz and Fz leads in two groups. (XLSX)

## Abbreviations

aERPs: auditory event-related potentials
ERP: event-related potential
